# Acute Primary Small Bowel Volvulus in a Male With Loeys-Dietz Syndrome

**DOI:** 10.7759/cureus.61956

**Published:** 2024-06-08

**Authors:** Sophia Yang, Rod Flynn, Tedla T Tessema

**Affiliations:** 1 Osteopathic Medicine, Liberty University College of Osteopathic Medicine, Lynchburg, USA; 2 General Surgery and Trauma, Mary Washington Healthcare, Fredericksburg, USA; 3 General Surgery, Mary Washington Healthcare, Fredericksburg, USA

**Keywords:** volvulus of midgut, acute volvulus, primary small bowel volvulus, loeys- dietz syndrome (lds), loeys-dietz syndrome

## Abstract

Loeys-Dietz syndrome (LDS) is a connective tissue disorder with features including, but not limited to, aortic dissections, skeletal abnormalities, and craniofacial defects. However, considering its relatively recent discovery, there are still many unknowns about LDS. The extent of a connective tissue disorder like LDS is yet to be defined throughout the various organ systems, including the gastrointestinal system. Connective tissue disorders have been found to have higher associations with certain conditions, like constipation. In a similar manner, LDS may increase the propensity for developing uncommon gastrointestinal manifestations, like primary small bowel volvulus. A volvulus is defined as an abnormal rotation of the small bowel segment along the axis of its mesentery. Primary small bowel volvulus is differentiated from secondary small bowel volvulus by its nature of origin: primary small bowel volvulus occurs as an independent spontaneous occurrence, whereas secondary small bowel volvulus is secondary to the presence of adhesions, diverticular disease, or abdominal masses. In this case report, we highlight a potential gastrointestinal manifestation of LDS with the occurrence of a primary small bowel volvulus in a young adult male diagnosed with LDS. The patient experienced acute primary small bowel volvulus 14 days into his stay, which may have been influenced by this newfound connective tissue disorder.

## Introduction

Loeys-Dietz syndrome (LDS) is a connective tissue disorder that alters the transforming growth factor beta (TGF-β) signaling pathway [1]. There are five types of LDS: Type 2 experiences more aggressive aortic disease; Type 3 has an increased prevalence of mitral valve prolapse and arthritis; and Types 4 and 5 experience fewer musculoskeletal and cardiovascular defects [2]. Some notable gastrointestinal manifestations include constipation, eosinophilic gastrointestinal disease, and inflammatory bowel disease [3].

However, an understanding of this connective tissue defect on a multisystem level is still being established today. Since there are still many unknowns regarding LDS, it is important to be aware of other potential manifestations of LDS.

In this case report, a 41-year-old male diagnosed with LDS presented with primary small bowel volvulus, which occurs at rates of 0.00001% to 0.19% [4]. We will discuss the patient’s admission and the course of hospital management leading up to the operation in which he was found to have a primary small bowel volvulus.

## Case presentation

We present the case of a 41-year-old Caucasian male residing at a long-term care facility for 10 months who was admitted to the ICU for acute respiratory failure and hypoxia after aspirating on a protein drink. One week later, a surgical consult was placed for a percutaneous esophagogastrostomy (PEG) tube. A CT scan of the abdomen and pelvis with IV contrast showed significant stool burden and a distended ascending and transverse colon with no signs of small bowel obstruction.

**Figure 1:**
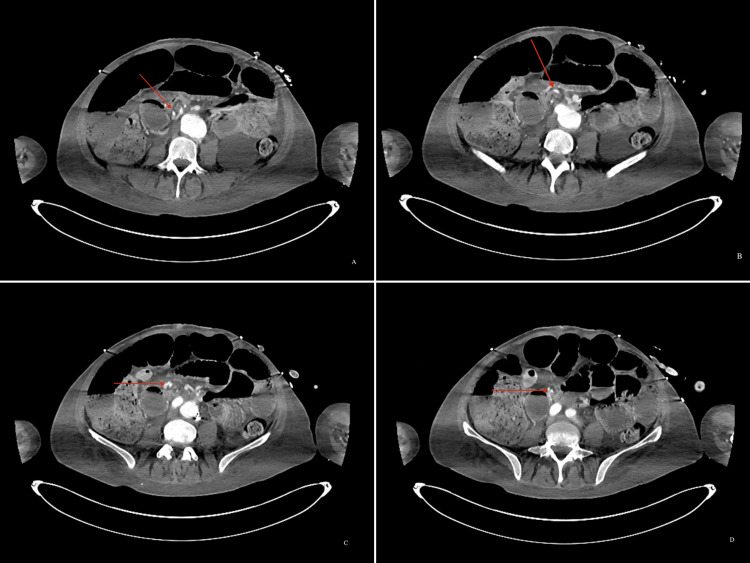
(A-D) Progression of the superior mesenteric artery in the “whirlpool sign”

On day 11, the patient underwent a percutaneous tracheostomy. The PEG was unsuccessful due to the lack of transillumination, so the operation was converted to an open gastrostomy. This operation revealed a distended transverse colon, likely secondary to severe constipation, but no dilated small bowel was found. Three days later, the patient was noted to be intolerant of tube feeding, and an abdominal X-ray was then performed, revealing a pattern of small bowel obstruction. A CT scan of the abdomen and pelvis with IV contrast was performed the following day, and a surgical consult was placed for the acute onset of small bowel obstruction secondary to internal herniation. The CT scan showed mesenteric swirling (Figure 1A-1D), the classic whirlpool sign, highly suspicious for acute small bowel volvulus, indicative of emergent exploratory laparotomy.

**Figure 2:**
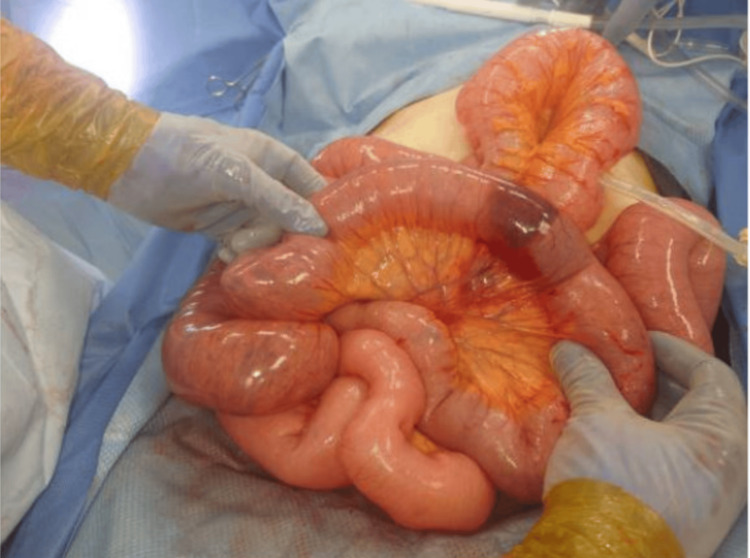
Hemorrhage in the small bowel

An exploratory laparotomy was performed less than three hours later. At laparotomy, a moderate amount of serous fluid and a massively distended small bowel were found. The small bowel was gently eviscerated with a relatively large dusky segment in the proximal ileum and a small bowel wall hemorrhage about 3 cm wide (Figure 2).

**Figure 3:**
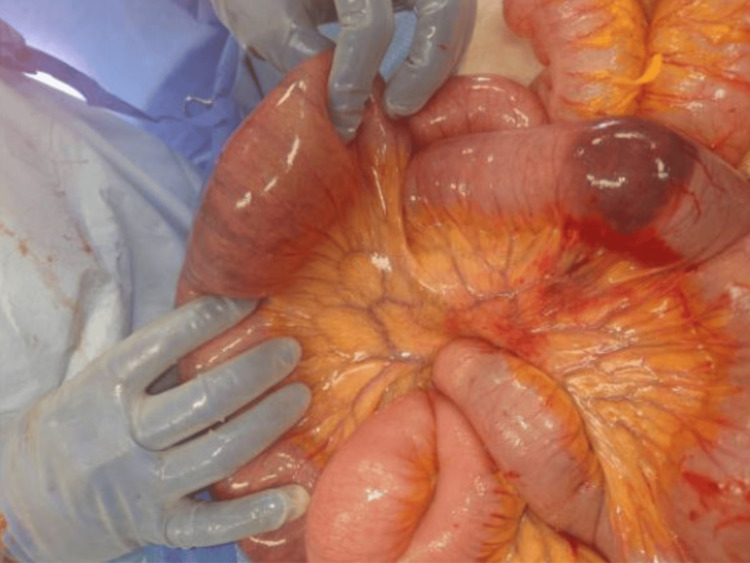
Abnormal rotation of the small bowel around the root of the mesentery

Abnormal rotation of the small bowel was noted at the root of the mesentery, which turned out to be a primary volvulus along its mesenteric axis (Figure 3). The rotation was in a clockwise pattern. There was no band around which the small bowel had rotated or any defect in the mesentery of the small bowel or the mesocolon to account for a secondary volvulus. Regarding the colon, the cecum and the ascending colon were massively dilated and filled with content from severe constipation. The entire colon, including the cecum, was in the normal anatomic location. The transverse colon was redundant and filled with a large amount of air, which was milked caudad toward the left colon and sigmoid colon. The sigmoid colon was normal in size.

**Figure 4:**
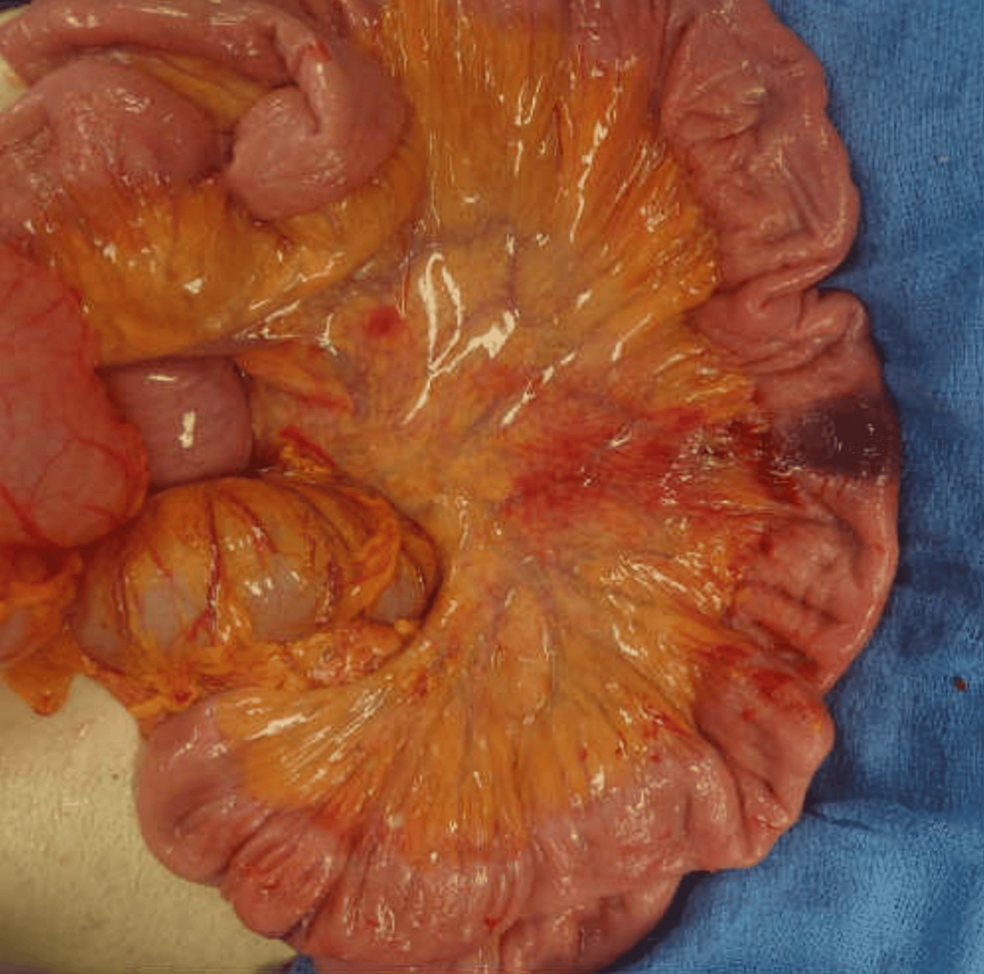
Decompressed small bowel with hemorrhage

An orogastric tube was placed, and the small bowel was decompressed through the stomach. Approximately 2 liters of enteric content was suctioned out, decompressing the entire small bowel. The dusky portion of the small bowel became pink. The segment of the bowel with the wall hemorrhage was viable and demonstrated peristalsis, as seen in Figure 4.

The patient’s abdomen was closed in standard fashion. He remained stable throughout the procedure and was transported back to the ICU in stable condition.

A CT scan of the abdomen and pelvis with IV contrast was performed one week after the operation, showing none of the dilated loops of the small bowel that were found the week prior. No evidence of small bowel obstruction was present. Once the patient was found to be stable, he was discharged from the ICU and transported to a long-term acute care facility.

## Discussion

This is the first known case report of the occurrence of small bowel volvulus in a young male with LDS.

Primary small bowel volvulus is defined as an intestinal rotation that occurs when there are no anatomical anomalies, which is in contrast to secondary small bowel volvulus that occurs due to adhesions, tumors, diverticula, malrotation, etc. Some predisposing factors of primary small bowel volvulus include unusual bowel mobility, abdominal wall laxity, decreased mesenteric fat, etc. [5]. Anatomical or dietary influences can induce situations where the bowel becomes hypermotile, hypermobile, or rapidly filled with large amounts of poorly digestible food, which increases the propensity of the primary small bowel volvulus [5]. In regions like Asia, Africa, and the Middle East, primary small bowel volvulus occurs at much higher rates of about 0.024-0.06% [6]. The significant difference in occurrence may be due to differences in diet, lifestyle, and physical fitness that influence the anatomical structure between these populations and, hence, the incidence of primary small bowel volvulus.

Primary small bowel volvulus is currently understood to be an uncommon occurrence in the general population of the United States of America [7]. In this case, the patient has a unique connective tissue disorder, LDS, that may be a contributing factor for primary small bowel volvulus in a 41-year-old Caucasian male.

One possible theory we propose as an explanation for a potential correlation between LDS and small bowel volvulus is that the affected TGF-β signaling pathway during embryonic development causes anatomic alterations of the gastrointestinal tract that predispose the patient to developing primary small bowel volvulus. TGF-β signaling pathways are extensively involved in the epithelial-to-mesenchymal transition during development, including the formation of the myocardium, atrioventricular endothelium, etc. [8], which explains the cardiovascular manifestations of LDS. Cecal volvulus, which is the second most common colonic volvulus, can occur in those who have a congenital mobile cecum secondary to the failed fusion of the mesentery with the parietal peritoneum [9]. Perhaps the defect in TGF-β signaling pathways is influencing the structural laxity of the mesentery, which may be a precipitating factor in the primary small bowel volvulus in this patient. Studies are still being conducted on the nature of the TGF-β signaling pathway in embryonic development, which may reveal a connection to gastrointestinal manifestations of LDS in the future. Further studies would be needed to test this hypothesis.

A second theory we propose is that people diagnosed with LDS experience higher rates of volvulus due to a greater tendency to experience constipation. Chronic constipation is one of the main causes of volvulus, along with abnormal bowel contents [10]. Other studies have compared gastrointestinal manifestations in LDS to other connective tissue disorders like Ehlers-Danlos. LDS was found to have a higher incidence of constipation and diarrhea out of the two disorders [11]. This patient was noted to have a significant amount of stool building up in both the small and large intestines, which may have been the cause for the onset of primary small bowel volvulus. An increased tendency for constipation among people diagnosed with connective tissue disorders, especially LDS, may incline this population toward the primary small bowel volvulus.

A third theory we would like to consider is that people affected by LDS simply have a combination of a physically thin body with a gut that is structured to be intolerant of a standard diet. In the management guidelines for LDS, it is recommended to monitor body mass index and have a low threshold for determining when to provide caloric supplementation. Such caloric supplementation, similar to a vegetarian diet with low protein, may contribute to an elongated bowel structure, as was discussed earlier at the beginning of the discussion. This elongated bowel may predispose them to primary small bowel volvulus.

It is noted that this may have been an incidental finding. However, we would like to propose that the nature of the connective tissue disorder is a predisposing factor that leads to the incidence of primary small bowel volvulus. This may mean that the primary small bowel volvulus could be identified as a gastrointestinal manifestation of LDS. Future studies could investigate whether small bowel obstruction secondary to primary small bowel volvulus occurs at a higher rate in patients with LDS than in the general public. Depending on the prevalence of primary small bowel volvulus among patients diagnosed with LDS, primary small bowel volvulus could potentially be a gastrointestinal manifestation of LDS.

## Conclusions

Based on this case, it may be beneficial to investigate whether primary small bowel volvulus is a frequent occurrence in people diagnosed with LDS, considering the novelty of this syndrome. We propose three possible theories as an explanation for how the connective tissue defect in LDS could potentially predispose patients toward small bowel volvulus. However, it is difficult to make definitive assertions due to the relatively low prevalence of this condition. There may be similarities or connections with other connective tissue disorders like Ehlers-Danlos that can lead to a deeper understanding of LDS. With further case reports and research on LDS, we expect greater awareness and definition of its potential gastrointestinal manifestations.
